# Maternal overprotection predicts consistent improvement of self-compassion during mindfulness-based intervention and existential approach: a secondary analysis of the EXMIND study

**DOI:** 10.1186/s40359-021-00521-w

**Published:** 2021-02-01

**Authors:** Nobuko Kawano, Takeshi Terao, Akari Sakai, Mari Akase, Koji Hatano, Masanao Shirahama, Hirofumi Hirakawa, Kentaro Kohno, Nobuyoshi Ishii

**Affiliations:** 1 Department of Neuropsychiatry, Faculty of Medicine, Oita University, Idaigaoka 1-1, Hasama-machi, Yufu-city, Oita 879-5593 Japan; 2 Department of Psychology, Faculty of Welfare and Health Sciences, Oita University, 700 Dannoharu, Oita-city, Oita 870-1192 Japan

**Keywords:** Psychotherapy, Mindfulness, Existential approach, Self-compassion, Maternal overprotection

## Abstract

**Background:**

Recently, we showed that 4-week mindfulness-based intervention (MBI) followed by 4-week existential approach (EXMIND) was as effective for developing self-compassion as 8-week MBI. This study aims to identify the predictors to EXMIND.

**Methods:**

Of the 63 participants who completed the EXMIND group, 60 participants had baseline, 4-week, and 8-week total scores of the Self-Compassion Scale (SCS). Of the participants, 49 were female and 11 were male, with a mean age of 48.4 years. We investigated the participants’ intervention response patterns, then used analysis of variance to compared those patterns by age, gender, and the baseline scores of the Temperament Evaluation of Memphis, Pisa and San Diego Auto-questionnaire, the Temperament and Character Inventory, Mini-Mental State Examination, the Japanese Adult Reading Test, Young Mania Rating Scale, Hamilton Rating Scale for Depression, the Parental Bonding Instrument, and the Purpose in Life Test. In addition, multivariate logistic regression analysis was performed to identify any response pattern predictors.

**Results:**

Participants were divided into 4 groups based on response patterns in the changes of total SCS scores of the EXMIND group. The first group consisted of 15 participants who responded positively to MBI, but negatively to the existential approach (A group). The second group consisted of 23 participants who responded negatively to MBI, but positively to the existential approach (B group). The third group consisted of 20 participants who responded positively to both MBI and the existential approach (C group). The fourth group consisted of only 2 participants who responded negatively to both MBI and the existential approach (D group). Participants who responded positively to both MBI and the existential approach (C group) reported more maternal overprotectiveness than the other participants (groups A, B, and D).

**Conclusions:**

The present findings suggest that maternal overprotection may predict consistent improvement of self-compassion during EXMIND therapy.

## Background

Mindfulness, which has been linked to various health benefits, is a method that teaches individuals how to regulate attention while applying non-judgmental acceptance, whereas the existential approach supports the uniqueness of each individual and helps with finding meaning in one’s life. Meta-analyses showed that mindfulness-based interventions (MBI) are moderately effective in reducing stress, depression, anxiety and distress and in ameliorating the quality of life of healthy individuals [1], and that MBI may help to improve psychological functioning in employees [2]. There is preliminary but insufficient evidence for self-compassion and psychological flexibility as mechanisms underlying mindfulness-based interventions [3].

Recently, we performed a randomized controlled trial to examine whether MBI and the existential approach could be combined sequentially, as well as whether they would operate antagonistically or cooperatively [4]. In that study, apparently healthy participants were first allocated to a waiting-list group, then subsequently assigned randomly to either 8-week MBI group or 4-week MBI group followed by 4-week existential approach (EXMIND) group.

With regard to how to perform EXMIND, the EXMIND consisted of weekly MBI for 4 weeks plus subsequent weekly existential approach for 4 weeks. The maximum number of participants per session was up to 23. It took 90–120 min for participants to complete each session. Just before each session, 30 min was allocated for them to fill out the questionnaires. No payment was required from participants.

During the first half (4 weeks), MBI sessions included raisin exercise, mindful breathing, body scan, walking meditation, and sitting meditation. Participants received explanatory notes at every session. Homework including both formal and informal training was encouraged for participants to train themselves for mindfulness reskilling.

During the second half (4 weeks), the EXMIND group started life-scan where participants observed their life from the past, present, and future. This method was based on Orbanic’s description of “The therapist assists distraught individuals in discovering meaningfulness, finding purpose, and actualizing self-love by facilitating deeper self-awareness, appreciation, and understanding of who they are, have been and their unique process of becoming. The therapist, guided by this view, helps a hopeless individual realize that as a ‘person being-in-time’, he or she is always living in limitless temporality where change is inevitable, and the future bestows hope”, which was based on Heidegger’s philosophy. In order to do so, we performed a life-scan where each individual was instructed to recall past successful (reasonable) experiences and their success was commended by actual working members, which probably brought about meaningfulness and pleasure of achieving purpose. Also, each individual was asked to recall past miserable (unreasonable) experiences, and their patience and perseverance were respected by the actual working members, which probably actualized self-love (or self-esteem). Moreover, thinking about the purpose and meaning of life not attributed to common sense but to their individual proper thought during individual interviews was considered to be the very existential, and considering that death awareness and contemplating it allows one to prioritize and decide what is important to a meaningful and creative experience for each individual life, confirming individual will to live better in the uncertain future during individual interviews was likely to be the very existential. Following the above notions, they continued to perform mindfulness as homework where they could include life-scan; at session 5, recalling past successful (reasonable) experiences and their success was commended during individual interviews; at session 6, recalling past miserable (unreasonable) experiences, and their patience and perseverance were respected during individual interviews; at session 7, thinking about the purpose and meaning of life not attributed to common sense but to their proper thought, accepting themselves as they are, and understanding that they are happy when they think so during individual interviews; at session 8, confirming individual will to live better in the uncertain future during individual interviews.

The primary outcome was Self-Compassion Scale (SCS) total scores taken at the beginning of the study (baseline), then at 4 weeks and 8 weeks, either during the intervention or while waiting. When viewing the results, both 8-week MBI and EXMIND groups showed a significant increase in SCS total scores, as compared to those in the waiting group, suggesting that MBI and an existential approach are not antagonistic, but may have cooperative effects. Furthermore, these results suggest that EXMIND may be a useful treatment.

As afore-mentioned in the previous paper [4], we had thought that MBI has static or passive components such as attentional control and non-judgmental acceptance, whereas the existential approach has dynamic or active components such as discovering meaningfulness, finding purpose, and actualizing self-esteem. In other words, it seems likely that MBI tries to accept the world as it is whereas existential approach attempts changing the world within each individual. Therefore, in our opinion, these two therapies may have opposite components and it is unclear whether combining MBI and existential approach sequentially would cause antagonistic effects or whether they operate in a cooperative manner.

Although the results were not antagonistic in the EXMIND as a whole [4], at the individual level, we confirmed that there were four different response patterns during the EXMIND (i.e., 4-week mindfulness-based intervention (MBI) followed by 4-week existential approach) by subtracting total Self-Compassion Scale scores at baseline from those at 4 weeks, and subtracting those at 4 weeks from those at 8 weeks individually: participants who responded positively to MBI, but negatively to the existential approach; participants who responded negatively to MBI, but positively to the existential approach; participants who responded positively to both MBI and the existential approach; and participants who responded negatively to both MBI and the existential approach. These were simple categorization of which threshold was 0 (0 or less than 0 were negative responses whereas more than 0 were positive responses).

With regard to predictors to MBI and/or EXMIND, high educational attainment predicted a decrease in depressive symptoms by MBI [5]. Although MBI was associated with significant increases in trait positive affect and momentary positive cognition [6], in a systematic review, two found no association between mindfulness interventions and cognitive function, two found improvement that was not sustained at the follow-up, and another two found sustained improvement at 2- or 6-months [7]. Besides intelligence, the quality of family relationships could affect mindfulness [8]. As for purpose in life (or life’s meaning), patients’ search for life’s meaning was the only significant predictor of willingness to participate in MBSR in cancer patients [6]. Also, cancer patients endorsing higher levels of dispositional mindfulness were more likely to pay attention to positive experiences, a tendency which was associated with positive reappraisal of stressful life events. Moreover, patients who engaged in more frequent positive reappraisal had a greater sense of meaning in life [9]. Regrettably, we could not find reports showing predictors to existential approach.

This study aims to identify the predictors of response patterns to EXMIND (weekly MBI for 4 weeks plus subsequent weekly existential approach for 4 weeks).

## Methods

This study secondarily used data from our previous study [4] where, in brief, we performed a randomized controlled trial consisting MBI group and EXMIND group at the city hall of Oita, Japan (Horuto-Hall Oita). Participants were recruited from October 1, 2016 and June 30, 2018. The Institutional Review Board of the Oita University Faculty of Medicine approved the trial on Sep 14, 2016 (number B16-023). All participants provided written informed consent. The protocols of the primary study [4] conformed to the provisions of the Declaration of Helsinki and were approved by the Oita University Ethics Committee. Since the purpose of the present study was included in those of the primary studies, secondary use of the data was assumed to be permitted.

In the present study, we focused on the EXMIND group. Of the 63 participants who completed the EXMIND group, 60 participants provided baseline, 4-week, and 8-week total scores for the SCS [10] consisting of six subscales (self-kindness, common humanity, mindfulness, self-judgment, isolation, and over-identification). Of these participants, 49 were female and 11 were male, and their mean age was 48.4 years old. As already mentioned, we divided them into four groups by their responses to MBI at 4 weeks by subtracting the baseline SCS total scores from the 4-week SCS total scores which were positive (i.e., improved) or negative (including 0; i.e., not improved), and the responses to the existential approach at 4 weeks by subtracting the 4-week SCS total scores from the 8-week SCS total scores, which were positive or negative (including 0).

The groups were compared using analysis of variance from the viewpoints of age, gender, and the baseline scores from the Temperament Evaluation of Memphis, Pisa and San Diego Auto-questionnaire (TEMPS-A) [11] which can evaluate dimensions of affective temperaments including cyclothymic, irritable, depressive, hyperthymic, and anxious temperament, the Temperament and Character Inventory (TCI) [12] including four dimensions of temperament such as novelty seeking, reward dependence, harm avoidance, and persistence, Mini-Mental State Examination (MMSE) [13] which can measure the subject’s orientation to time and place, as well as the subject’s attention, memory, and computing power, the Japanese Adult Reading Test with 50 Kanji compound words (JART) [14] estimating current Intelligence Quotient (IQ) from the reading performance of *Kanji* compound words, Young Mania Rating Scale (YMRS)[15] which can assess manic or hypomanic state, Hamilton Rating Scale for Depression (HRSD) [16] which can assess depressive state, the Parental Bonding Instrument (PBI) [17] containing four subscales including paternal care, paternal overprotection, maternal care, and maternal overprotection, and the Purpose in Life Test (PIL) [18] which can assess the general sense of meaning and purpose in life. All the assessment tools were administered to the participants directly before the EXMIND intervention because they seemed to be associated with the responses to EXMIND. Finally, multivariate logistic regression analysis was performed to identify the predictors of intervention response patterns. If necessary, further post hoc analyses were performed to clarify the findings obtained through the above procedures.

## Results

There were four groups of intervention response patterns based on the changes of the total SCS scores of the EXMIND group. The first group consisted of 15 participants who responded positively to MBI, but responded negatively to the existential approach (A group). The second group consisted of 23 participants who responded negatively to MBI, but responded positively to the existential approach (B group). The third group consisted of 20 participants who responded positively to both MBI and the existential approach (C group). The fourth group consisted of only 2 participants who responded negatively to both MBI and the existential approach (D group). D group consisted of a 54-year-old female and a 41-year-old female, and their total SCS cores were 21.8 and 20.3 at the baseline 19.5 and 18.4 at 4 weeks, and 18.5 and 17.7 at 8 weeks, respectively. Thus, their score changes from the baseline to 4 weeks were −2.3 and − 1.9 and from 4 to 8 weeks were − 1.0 and − 0.7, respectively. To investigate the validity of the categorization, we compared their total responses to MBI and existential approach from baseline to 8 weeks by subtracting the baseline SCS total scores from the 8-week SCS total scores between A, B, C, and D groups using ANOVA with Tukey's post hoc test. As a result, there was a significant difference (F = 11.1, *p* < 0.0001) and the mean of the changes were 0.9 (A group which responded positively to MBI, but responded negatively to the existential approach), 1.4 (B group which responded negatively to MBI, but responded positively to the existential approach), 4.3 (C group which responded positively to both MBI and the existential approach), − 3 (D group which responded negatively to both MBI and the existential approach). C group had the significantly largest improvement than the other three groups, supporting the validity of the categorization. Nonetheless, the size of D group was too small; therefore, these two participants were excluded from the following analyses.

**Fig. 1:**
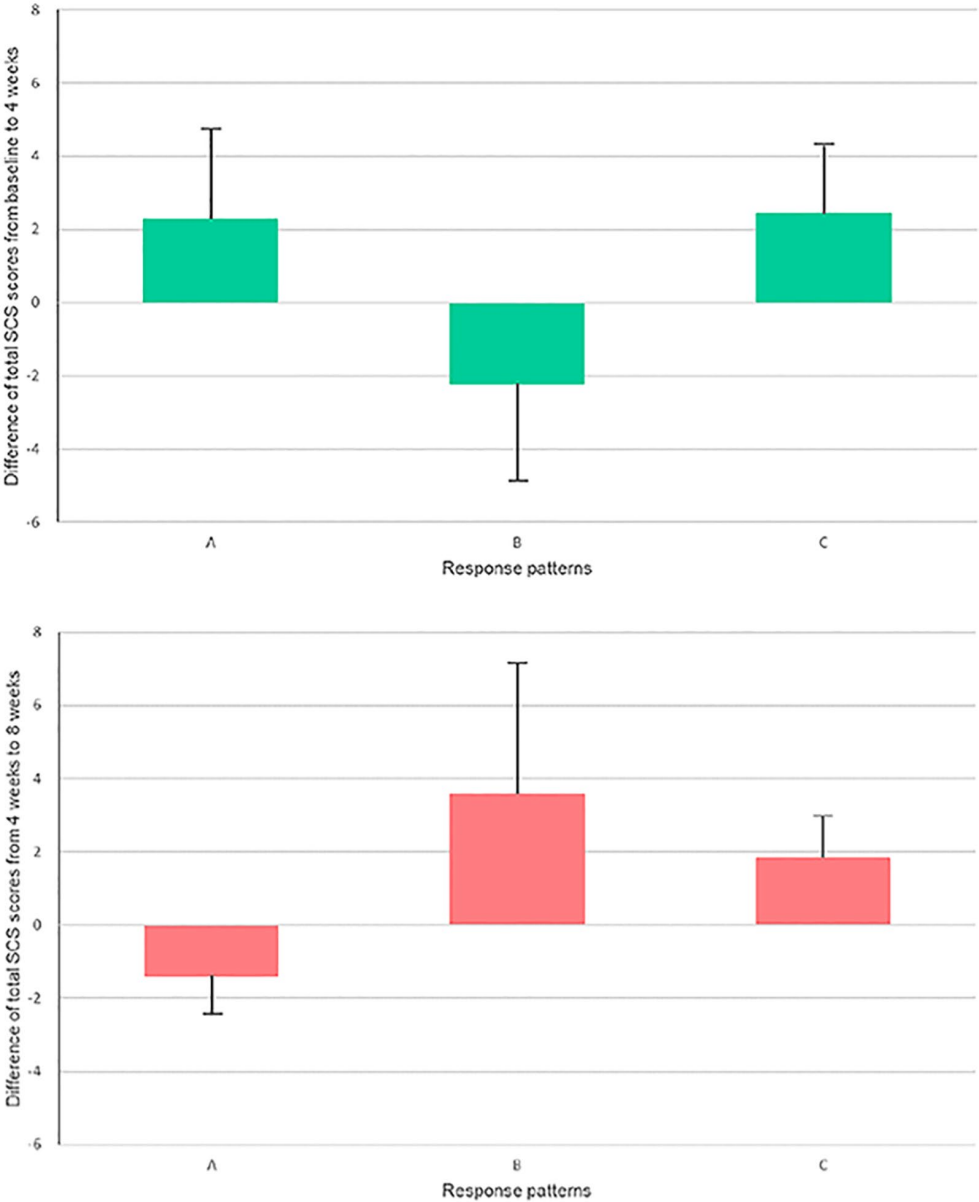
Changes in total Self-Compassion Scale (SCS) scores for each group. According to the intervention response patterns, there was A group (responded positively to the 4-week mindfulness-based intervention, but negatively to the 4-week existential approach); B group (responded negatively to the 4-week mindfulness-based intervention, but positively to the 4-week existential approach); and C group (responded positively to both the 4-week mindfulness-based intervention and the 4-week existential approach). D group (responded negatively to both the 4-week mindfulness-based intervention and the 4-week existential approach) was not included in this figure

As for the remaining three groups, the means of the total baseline SCS scores were 19.0 (SD = 5.1) for A group, 20.0 (SD = 4.2) for B group, and 16.5 (SD = 3.5) for C group (F = 2.99, *p* = 0.06). Figure 1 shows the changes in total SCS scores from the baseline to 4 weeks and the changes from 4 to 8 weeks for the three remaining groups. The mean improvement of A group from the baseline to 4 weeks was 2.3 and from 4 to 8 weeks it was − 1.4. The mean improvement of B group from the baseline to 4 weeks was—2.2 and from 4 to 8 weeks it was 3.6. The mean improvement of C group from the baseline to 4 weeks was 2.4 and from 4 to 8 weeks it was 1.8.

Table 1 shows the demographic variables and baseline scores of the three groups. Among the groups, the baseline scores of IQ estimated by the JART and reported maternal overprotection (as measured by the PBI) are significantly different and age is nearly significantly different; however, the other factors are not significantly different. Results from Tukey’s post hoc analyses show that A group has significantly higher IQ (JART) scores than B group, and that C group reported significantly higher rates of maternal overprotection in childhood than groups A and B.

**Table 1:** Demographic variables and baseline scores of psychological tests

	A group (n = 15)	B group (n = 23)	C group (n = 20)	Statistic	*p* value	Post-hoc comparison
Age	45.2 (14.7)	46.4 (12.8)	53.9 (10.8)	*F* = 2.61	0.08	
Sex				χ^2^ = 1.12	0.57	
Male	4 (27%)	3 (13%)	4 (20%)			
Female	11 (73%)	20 (87%)	16 (80%)			
TEMPS-A						
Depressive	7.1 (3.7)	8.6 (3.5)	8.8 (3.9)	*F* = 0.92	0.41	
Cyclothymic	3.4 (2.8)	6.1 (4.7)	5.5 (5.2)	*F* = *1.55*	0.22	
Hyperthymic	6.9 (4.0)	5.5 (3.6)	6.3 (4.0)	*F* = 0.58	0.56	
Irritable	2.2 (2.3)	3.4 (3.7)	3.3 (3.0)	*F* = 0.62	0.54	
Anxious	6.7 (6.0)	7.1 (6.0)	6.6 (4.8)	*F* = 0.06	0.94	
TCI						
Novelty Seeking	19.9 (3.8)	21.4 (5.6)	21.9 (4.2)	*F* = 0.85	0.43	
Harm Avoidance	18.3 (8.0)	18.3 (8.2)	18.3 (5.7)	*F* = 0.00	1.00	
Reward Dependence	14.5 (3.5)	15.1 (3.3)	15.2 (4.8)	*F* = 0.16	0.85	
Persistence	4.6 (2.5)	4.5 (1.7)	4.5 (2.0)	*F* = 0.02	0.98	
Self-Directedness	25.3 (11.6)	28.9 (7.6)	27.6 (8.7)	*F* = 0.72	0.49	
Cooperativeness	27.0 (7.7)	29.4 (3.8)	26.2 (7.4)	*F* = 1.48	0.24	
Self-Transcendence	13.5 (4.9)	12.9 (6.2)	15.0 (6.0)	*F* = 0.68	0.51	
MMSE	29.3 (0.8)	29.6 (0.6)	29.3 (0.7)	*F* = 0.89	0.42	
IQ (JART)	114.5 (5.6)	107.4 (7.0)	109.7 (6.9)	*F* = 5.32	0.01	A > B^a^
YMRS	0.4 (0.7)	0.2 (0.6)	0.3 (0.8)	*F* = 0.33	0.72	
HRSD	1.3 (2.3)	2.3 (2.4)	2.6 (2.9)	*F* = 1.13	0.33	
PBI						
Paternal Care	20.6 (8.7)	22.6 (7.8)	21.3 (7.4)	*F* = 0.28	0.76	
Paternal overprotection	10.4 (6.9)	10.6 (5.8)	12.3 (6.6)	*F* = 0.49	0.61	
Maternal Care	26.5 (6.6)	24.3 (5.7)	23.9 (7.5)	*F* = 0.71	0.50	
Maternal overprotection	10.6 (6.8)	11.3 (7.4)	17.0 (7.9)	*F* = 4.32	0.02	A,B < C^b^
T-score (PIL)	47.5 (10.5)	48.1 (8.7)	50.3 (11.4)	*F* = 0.38	0.69	

Data are mean (SD) or number (%) unless otherwise indicated

A group: Participants who responded positively to MBI, but negatively to the existential approach. B group: Participants who responded negatively to MBI, but positively to the existential approach. C group: Participants who responded positively to both MBI and existential approach

^a^A vs. B: *p* = 0.01, A vs. C: *p* = 0.09, B vs. C: *p* = 0.51

^b^A vs. B: *p* = 0.96, A vs. C: *p* = 0.04, B vs. C: *p* = 0.04

Abbreviations: TEMPS-A, Temperament Evaluation of Memphis, Pisa and San Diego Auto-questionnaire; TCI, Temperament and Character Inventory; MMSE, Mini-Mental State Examination; IQ, Intelligence Quotient; JART, Japanese Adult Reading Test; YMRS, Young Mania Rating Scale; HRSD, Hamilton Rating Scale for Depression; PBI, Parental Bonding Instrument; PIL, Purpose in Life Test

Multivariate logistic regression analyses of response patterns (groups A, B, and C themselves) as a dependent factor (C group as a reference) with age, IQ (JART), and rates of childhood maternal overprotection from the PBI as independent factors reveal a significantly positive association with IQ (JART) and significantly negative associations with age and reported maternal overprotection (PBI) in A group when compared to C group, and a significantly negative association with reported maternal overprotection (PBI) in B group when compared to C group (Table 2).

**Table 2:** Multivariate logistic regression analyses

	Odds ratio (95% Cl)	*P* value
A group (n = 15)		
Intercept		0.156
Age	0.93 (0.87–0.99)	0.021
IQ (JART)	1.17 (1.00–1.38)	0.048
Maternal overprotection (PBI)	0.89 (0.80–0.99)	0.030
B group (n = 23)		
Intercept		0.082
Age	0.95 (0.89–1.00)	0.058
IQ (JART)	0.95 (0.86–1.05)	0.323
Maternal overprotection (PBI)	0.89 (0.81–0.97)	0.011
Reference: C group (n = 20)		

The above findings of the rates of reported maternal overprotection in childhood (PBI) being significantly higher in C group than in groups A and B led us to perform the following post hoc analyses. We analyzed the association between reported maternal overprotection (PBI) and baseline total SCS scores for 58 participants (groups A, B, and C), and the results showed that the association was significantly negative (r = − 0.438, *p* = 0.001). Moreover, we analyzed the association between baseline total SCS scores and the changes in total SCS scores for these 58 participants, which showed that the association was significantly negative (r = − 0.319, *p* = 0.015).

## Discussion

The findings of the present study show four intervention response patterns in the EXMIND (4-week MBI group followed by 4-week existential approach) group, although only two participants responded negatively to both MBI and the existential approach. The remaining participants were categorized into A group (responded positively to MBI, but negatively to the existential approach), B group (responded negatively to MBI, but positively to the existential approach), and C group (responded positively to both MBI and the existential approach).

Interestingly, rates of reported maternal overprotection in childhood were significantly higher in C group than in groups A and B, suggesting that participants with positive responses to both MBI and existential approach experienced more past maternal overprotection. The post hoc analyses showed significantly negative associations between reported maternal overprotection (PBI) and baseline total SCS scores and between baseline total SCS scores and changes in total SCS scores. This indicates that experiencing maternal overprotection in childhood may lead to decreased self-compassion, while EXMIND therapy could help to increase self-compassion. As such, reported maternal overprotection in childhood may be able to predict consistent positive responses to both MBI and the existential approach during EXMIND therapy.

Asano et al. [19] showed that experiencing lower maternal overprotection in childhood was significantly associated with better responses to cognitive behavior therapy (CBT) for individuals with depression. They posited that reporting lower maternal overprotection indicates experiencing a less controlling maternal parenting style in childhood. Subsequently, experiencing a less controlling maternal parenting style in childhood may generate a better relationship between individual and therapist, which may then produce a better treatment outcome. These findings suggest that lower reported maternal overprotection in childhood may predict a better outcome of CBT. Furthermore, Johnstone et al. [20] demonstrated that among individuals with depression receiving interpersonal psychotherapy (IPT), only 25% of those individuals who reported high childhood paternal protection (overprotection) responded positively to IPT, whereas 60% of the individuals who reported low or intermediate childhood paternal protection responded positively to IPT. Additionally, individuals who reported low and high maternal care in childhood responded less well to IPT (29% and 48% positive response rate, respectively), whereas 80% of individuals who reported intermediate maternal care in childhood responded positively to IPT. The care dimension measures the informant’s perception of affection and warmth expressed by the parent toward the offspring, whereas the control dimension measures the extent of a parent’s overprotection and authoritarianism (i.e., excessive interference with the offspring’s autonomy) [21]. These findings indicate that low or intermediate paternal protection and/or intermediate maternal care in childhood may predict better outcomes for IPT.

Our results were different from these previous findings. One of the reasons may be that our EXMIND therapy is different from CBT or IPT. Presumably, existential therapy may compensate for the immature part of the self, which could have been influenced by maternal overprotection in childhood. Therefore, within the context of EXMIND therapy, maternal overprotection in childhood could be an indicator of a positive response to the intervention. This can be applied to B group (responded negatively to MBI, but positively to the existential approach) and C group (responded positively to both MBI and the existential approach).

With regard to the mechanism of action of EXMIND (MBI and existential approach), MBI teaches individuals how to regulate attention while applying non-judgmental acceptance and thereafter existential approach supports the uniqueness of each individual and helps with finding meaning in one’s life particularly in the individuals with maternal overprotection in childhood. Probably, MBI make individuals objectively think their thoughts by themselves and on such firm ground they can foster self-compassion. Moreover, via existential approach, they can find meaning in their lives with enhancement of self-compassion. Toyoshima et al. [22, 23] showed that the relationship between childhood parental bonding such as maternal overprotection and lower adulthood cognitive function was mediated with affective temperaments and depressive symptoms, and that the relationship between childhood parenting such as maltreatment and lower adulthood social function was mediated with depressive symptoms and cognitive function. These findings suggest the interaction between childhood parental bonding/parenting, affective temperaments, depressive symptoms, cognitive complaints and social function in adult community volunteers, and particularly the negative effect of maternal overprotection on affective temperaments, depressive symptoms, cognitive complaints and social function, which may be associated with adult low self-compassion.

It should be noted that as one of predictors, maternal overprotection as indicated by the PBI can ostensibly be used in participant selection for either CBT/IPT or EXMIND. That is, participants who experienced low maternal overprotection in childhood may respond well to CBT/IPT, whereas participants who experienced high maternal overprotection in childhood may respond well to EXMIND. Moreover, there is a report showing that greater perceived criticism and lower perceived praise predict schizotypy in the healthy population [24]. Schizotypy is defined as 'a latent personality organization reflecting a putative liability for schizophrenia-spectrum disorders and the psychoses [25]. Therefore, it seems likely that healthy individuals with schizotypy may respond to EXMIND. Further studies are, however, required to substantiate these possibilities.

With regard to the other predictors, A group, but not B group, had significantly younger ages and higher IQ (JART) scores than C group. This is most likely because their younger age may be indicative of fewer life experiences, making the existential approach less accessible, while higher IQ scores may lead them to examine their lives objectively rather than subjectively and make them less think of themselves as proper ones in an existential way. Just in case, the validity of the JART has been established in a study with fifty normal elderly people in which their JART scores were significantly and positively associated with all the subscale scores of WAIS-R [26].

As for the two participants in group D, we could not identify a reason as to why they responded negatively to both MBI and the existential approach, although one had experienced significant past trauma. Interestingly, their maternal overprotection scores from the PBI were very low (0 and 5). Therefore, not only A group and B group, but also D group, had lower maternal overprotection scores from the PBI than C group.

The limitations of this study are: (1) a relatively small sample size with a higher percentage of a mainly middle-age, female, educated and employed group of individuals, (2) a recall error or bias owing to the nature of questionnaires such as the PBI, (3) not including individuals with psychiatric disorders, which preclude us from predicting the effects of EXMIND therapy for individuals with psychiatric disorders, and (4) the post hoc study design. Finally, the subjects were limited to Japanese, which precludes generalization of the present findings.

## Conclusions

The present findings suggest that experiencing maternal overprotection in childhood may predict consistent improvement of self-compassion during EXMIND therapy. Here, “consistent improvement” means improvement in both 4-week mindfulness-based intervention (MBI) and subsequent 4-week existential approach.

## Data Availability

The datasets used and analyzed during the current study are available from the corresponding author on reasonable request.

## Abbreviations

CBT: cognitive behavior therapy
EXMIND: 4-week mindfulness-based intervention followed by 4-week existential approach
HRSD: Hamilton Rating Scale for Depression
IPT: interpersonal psychotherapy
IQ: Intelligence Quotient
JART: Japanese Adult Reading Test with 50 Kanji compound words
MBI: mindfulness-based intervention
MMSE: Mini-Mental State Examination
PBI: Parental Bonding Instrument
PIL: Purpose in Life Test
SCS: Self-Compassion Scale
TCI: Temperament and Character Inventory
TEMPS-A: Temperament Evaluation of Memphis, Pisa and San Diego Auto-questionnaire
YMRS: Young Mania Rating Scale
